# Coordinated Human Brainstem and Spinal Cord Networks during the Expectation of Pain Have Elements Unique from Resting-State Effects

**DOI:** 10.3390/brainsci10090568

**Published:** 2020-08-19

**Authors:** Gabriela Ioachim, Jocelyn M. Powers, Howard J. M. Warren, Patrick W. Stroman

**Affiliations:** 1 Centre for Neuroscience Studies, Queen’s University, Kingston, ON K7L 3N6, Canada; 16gi@queensu.ca (G.I.); jocelyn.powers@queensu.ca (J.M.P.); howie.warren@queensu.ca (H.J.M.W.); 2 Department of Biomedical and Molecular Sciences, Queen’s University, Kingston, ON K7L 3N6, Canada; 3 Department of Physics, Queen’s University, Kingston, ON K7L 3N6, Canada

**Keywords:** pain, human, spinal cord fMRI, connectivity networks

## Abstract

Functional magnetic resonance imaging (fMRI) research on the human brainstem (BS) and spinal cord (SC) has identified extensive BS/SC resting-state networks (RSNs) by showing spontaneous coordinated blood oxygenation-level dependent (BOLD) signal fluctuations in the absence of a stimulus. Studies have shown that these networks can be influenced by participants’ level of arousal or attention (e.g., watching a video), and linked network function to autonomic homeostatic regulation. Here we explore how the cognitive state of expecting pain can influence connectivity in these networks. Data from two studies (a predictable pain stimulus study, and a resting-state study) were compared to show the effects of expecting pain on BS/SC networks, and how networks differed from networks associated with the resting-state. In each study, BOLD fMRI data were obtained from the cervical SC and brainstem in healthy participants at 3 tesla using a T_2_-weighted single-shot fast spin-echo imaging method. Functional connectivity was investigated within the entire 3D volume by means of structural equation modeling (SEM) and analyses of covariance (ANCOVA). Results showed extensive connectivity within/across BS and SC regions during the expectation of pain, and ANCOVA analyses showed that connectivity in specific components of these networks varied with individual pain sensitivity. Comparing these results to RSN fluctuations revealed commonalities in coordination between BS and SC regions, and specific BS–BS connectivity fluctuations unique to the expectation of pain. Based on the regions involved, these results provide evidence of brainstem regulation specific to the expectation of pain.

## 1. Introduction

The spinal cord (SC) plays a significant role in sensory and motor functions and functional magnetic resonance imaging (fMRI) has expanded our understanding of its function. Studies to date have demonstrated that the SC is not simply a relay for neural signaling between the periphery and brain, but contributes to complex processes such as sensory and pain regulation [1]. In particular, studies of pain processing have demonstrated signaling within/between SC and brainstem (BS) regions in the absence of a stimulus, such as during the anticipation of pain [1,2]. In the past decade, several groups have also described resting-state blood oxygenation-level dependent (BOLD) signal fluctuations in the spinal cord that occur in the absence of any external stimulus, and have concluded that these likely represent coordinated resting-state networks (RSNs) [3,4,5,6,7]. While the extent and specific function of these networks are only beginning to be explored, recent studies have greatly expanded our understanding of coordinated activity across the cervical SC and BS [7,8,9].

Tonic SC input from specific regions of the BS has been shown to regulate sensory and pain signaling [10], and many of these BS regions have also been shown to be important components of the recently-described RSNs [7,8]. This also suggests that the spinal cord is in an active state, regulated by brainstem regions, even during a ‘baseline’ state of an fMRI study when no stimulus (such as pain) is being applied. Further supporting this idea, a recent analysis of data from 59 participants in previous pain studies showed that BOLD signal variations in specific areas of the BS/SC were associated with participants’ expectations of upcoming painful stimuli [1]. This study identified BS/SC networks that involve autonomic homeostatic regulation and descending pain modulation. In a separate study we also compared a resting-state condition to periods when participants were engaged in watching a video or listening to an audio presentation [8]. The results confirmed that watching a video or listening to music altered connectivity in previously identified BS/SC RSNs, and included regions involved with autonomic and homeostatic regulation [8]. Collectively, this body of research shows that coordinated SC and BS networks perform important regulatory functions even before the administration of a noxious stimulus. 

The purpose of the current study was to investigate the effects of expectation of pain on brainstem and spinal cord regions known to be involved in resting-state networks. Based on the research to date, we hypothesize that network connectivity in the spinal cord and brainstem during the expectation of pain will involve components of previously identified resting-state networks. While this cannot be considered a ‘resting-state’ study as the expectation of pain is a manipulation of a participant’s cognitive and emotional state, the results of this study could provide important insights into our understanding of how coordinated spinal cord and brainstem networks serve to integrate autonomic regulation with pain processing.

## 2. Materials and Methods

All methods were approved by the institutional human research ethics review board. All participants were healthy adults and informed consent was obtained in writing from each participant before onset of the study. It was approved by the Queen’s University Health Sciences & Affiliated Teaching Hospitals Research Ethics Board, TRAQ# 6015383.

### 2.1. Participants and Experimental Setup

The current study incorporates data from two previously published BS/SC fMRI studies. As both studies are described elsewhere [2,8] only the details relevant to the current study are discussed here.

#### 2.1.1. Predictable Pain Data

A prior functional MRI study involving a predictable noxious heat stimulus was conducted with 17 healthy participants [2]. Functional imaging data spanning the brainstem and cervical spinal cord were collected and used for the current analysis. The 17 participants (13 women, 4 men, aged 22 ± 3 years) were recruited through online and paper advertisements in the local community. They were free from any neurological disorders, major illnesses, psychiatric disorders, or MRI contraindications (such as metallic implants or pacemakers). All participants underwent a 1 h sham MRI training session (which included quantitative sensory testing) and an imaging session of their brainstem and cervical spinal cord.

The heat stimuli were administered using an MRI-compatible Peltier thermode (Medoc^®^, Ramat Yishai, Israel) which was set to a constant temperature calibrated to elicit moderate pain for each individual. This calibration was carried out during the quantitative sensory testing portion of training, where participants were also familiarized with a 100-point pain intensity rating scale [11] with verbal descriptors at each 10-point interval (0 = no sensation, 10 = warm, 20 = a barely painful sensation, 30 = very weak pain, 40 = weak pain, 50 = moderate pain, 60 = slightly strong pain, 70 = strong pain, 80 = very strong pain, 90 = nearly intolerable pain, 100 = intolerable pain). An experimenter held the thermode and applied it repeatedly to the skin of the participant’s right hand (overlaying the thenar eminence and corresponding to the sixth cervical cord segment dermatome). The experimenter was cued through audio signals to deliver the contacts at precise timings during the experiment. While quantitative sensory testing for pain thresholds has limitations as pain is very subjective [12] and can vary with the modality of pain applied [13], this particular session aimed to establish a temperature for each individual that would elicit a consistent response of moderate pain which was used in the subsequent fMRI portion of the study.

**Figure 1:**
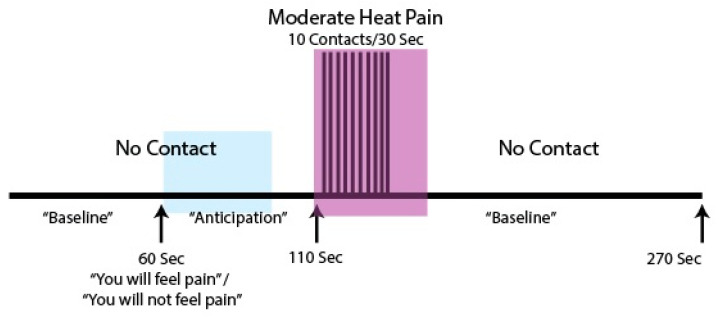
Task paradigm for the expecting pain data. Highlighted areas indicate periods analyzed in the current study, with the blue highlighting indicating the ‘expectation’ period (after participants were told whether or not a painful stimulus would be applied) and purple highlighting indicating the ‘stimulation’ period (where participants were either experiencing a painful stimulus, or not experiencing a stimulus.

The stimulation paradigm (Figure 1) spanned 4 min and 20 s and consisted of 1 min 50 s of a baseline period with no contacts, then 10 heat contacts delivered over 30 s, followed by 2 min of no stimulation.

Participants underwent 10 functional imaging runs, 5 in which the heat stimulus was applied and 5 in which there was no stimulation, with the run types varied in a randomized order. One minute into each run, participants were told whether or not they would be feeling a heat stimulus, and could therefore anticipate the painful stimulus for the second minute until the heat contacts began at 1 min 50 s into the run. In between each run, participants were given 2 min of rest time to avoid sensitization of nociceptors in the skin. During this rest time, the experimenter obtained the participants’ verbal ratings of their pain intensity for the first and last heat contact of that run. Importantly for the current study, participants could anticipate whether or not there would be pain involved in each run, and could anticipate the timing of the stimulus and baseline periods.

#### 2.1.2. Resting State Data

Functional MRI data from a second previous study [8] were used for comparison with the predictable pain data. Data were collected from 20 healthy participants (14 women, 6 men, aged 21 ± 2 years) who were free from any neurological disorders, major health conditions, or MRI contra-indications. These participants were instructed to lie still in the scanner and were told they may be listening to audio, watching a video, or seeing a picture, but were given no specific task to carry out. The resting-state (‘picture’) condition consisted of a presentation of a landscape picture, while the ‘audio’ condition consisted of a presentation of a piece of music and a piece of spoken word poetry, each in different runs. Lastly, the ‘video’ condition consisted of a presentation of a card magic trick and a video game demonstration, each in different runs. The details of these presentations and setup are described in a previous publication. Each condition presentation was 6 min and 57 s long, and each participant experienced two of each condition presentations, in a random order, for a total of 6 functional scans.

### 2.2. Functional MRI Data Acquisition

The full setup for the prior studies is described in detail elsewhere [2,8], therefore only the details relevant to the current analysis are discussed here. The ‘predictable pain’ and ‘resting state’ data were collected with the same acquisition method and imaging parameters. Functional MRI scans were carried out on a Siemens 3 tesla MRI system (Siemens Magnetom Trio, Erlangen, Germany). Localizer images were acquired in three planes to provide a reference for subsequent slice positioning. Images of the full brainstem and cervical spinal cord were acquired using a half-Fourier single-shot fast spin-echo sequence (HASTE) with BOLD contrast [14]. This method has been shown to provide optimal image quality and BOLD sensitivity in the brainstem and spinal cord [1]. The 3D volume spanned from the first thoracic vertebra to above the thalamus and was imaged in 9 contiguous sagittal slices, with a 28 × 21 cm field-of-view and 1.5 × 1.5 × 2 mm^2^ resolution. Imaging parameters included an echo time (TE) of 76 m and a repetition time (TR) of 6.75 s/volume for optimal T_2_-weighted BOLD sensitivity and for consistency with our previous studies [15,16,17,18,19].

**Figure 2:**
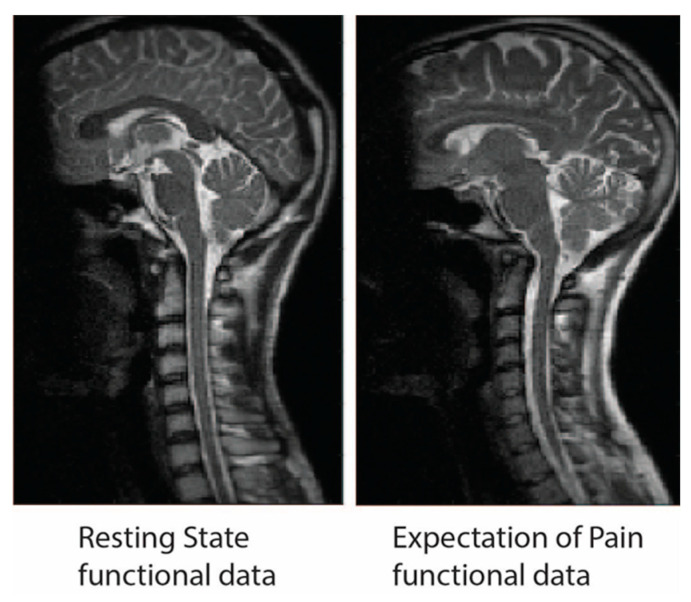
Examples of sagittal-slice functional data from an individual in the ‘resting state’ and an individual in the ‘expectation of pain’ data set for comparison. As both studies collected data with the same methods on the same scanner, the quality of the two data sets is equivalent.

For the ‘predictable pain’ data, each condition consisted of 40 volumes acquired per run (200 volumes total per condition, over 5 repeated runs). In total, 10 runs were acquired for each participant (5 with the heat stimulus and 5 without). For the ‘resting state’ data, each condition consisted of a total of 62 volumes acquired to produce a time-series spanning 6 min 57 s. In total, 6 runs were acquired for each participant (2 Picture, 2 Audio, and 2 Video). Examples of functional data from each data set are available in Figure 2.

### 2.3. Data Preprocessing

Data preprocessing was carried out in the same manner for both the ‘predictable pain’ and ‘resting state’ data sets. Spinal cord and brainstem fMRI data were preprocessed and analyzed using custom-written software, “spinalfmri8” (http://post.queensu.ca/~stromanp/software.html) in MATLAB (MathWorks, Natick, MA, USA), which is the most widely used analysis software for spinal cord fMRI [1]. Images were converted to NIfTI format and co-registered to correct for bulk body motion using the non-rigid 3D registration tool in the MIRT (Medical Image Registration Toolbox) package [20,21]. The motion parameters obtained from the co-registration procedure were used as models of bulk movement, and later used for removing/reducing noise in the data. The images were resized to 1 mm^3^ voxels and spatially normalized to a predefined anatomical template based on 356 participants, by means of an automated procedure which has been described previously [2,18]. Physiological noise estimates were also obtained from the recording of the peripheral pulse (which was synchronized to each fMRI time series) and estimates of global noise were obtained from predefined regions of white matter. The noise models (bulk motion, cardiac-related, and white matter) were fitted to the data using a general linear model (GLM), and then subtracted from the data. This method has been shown previously to be effective at removing physiological noise [15].

#### 2.3.1. Predictable Pain Data

For the current study, the second minute of the baseline period preceding the noxious stimulus as well as the stimulation period were analyzed. The volumes analyzed during the second minute spanned up to 1 min 51 s into a run. While this time period does include 1 s during the stimulation period, this does not include any BOLD responses that can be attributed to the stimulus as the hemodynamic response is slow, needing roughly 2 s to begin rising, and 5–6 s to reach its peak after the stimulus onset [22]. This resulted in two groups of data to be analyzed in the ‘expectation’ period (before a stimulus was applied): periods during which the participants were expecting pain (‘expect pain’), and periods in which the participants were expecting no pain (‘expect no pain’), and two groups of data to be analyzed in the ‘stimulation’ period: stimulation periods during which the participants were experiencing pain (‘pain’), and corresponding periods in which participants were not experiencing the heat contacts (‘no pain’). This was done in order to be able to compare results to previous spinal fMRI resting-state studies as well as our recent study examining how these networks may be affected by participants experiencing different cognitive states [7,8].

#### 2.3.2. Resting State Data

To provide the best comparison to the ‘predictable pain’ data, analysis of the ‘resting-state’ data focused on the same time periods above (between 1 min and 2 min to compare to the ‘expectation’ period of the ‘predictable pain’ data, and between 2 min and 2 min 30 s to compare to the ‘stimulation’ period). This ensured that confounds such as participant fatigue after a number of minutes into a run were not interfering with any conclusions drawn from these comparisons.

### 2.4. Data Analysis

Both the ‘predictable pain’ and ‘resting state’ data sets were analyzed with the same process. Therefore, overall data analysis is described here, with details specific to each data set described when relevant.

Data were averaged over clusters of voxels to reduce the number of statistical comparisons to be made and increase the signal-to-noise ratio over that of single-voxel analyses. First, we identified 10 Regions of Interest (ROIs) using a previously-established anatomical region map [16,18,23], which included the hypothalamus, periaqueductal gray matter (PAG), parabrachial nucleus (PBN), locus coeruleus (LC), nucleus tractus solitarius (NTS), nucleus raphe magnus (NRM), nucleus gigantocellularis (NGc), dorsal reticular nucleus of the medulla (DRt), pontine reticular formation (PRF), and the right dorsal quadrant of the sixth cervical spinal cord segment (C6RD). This spinal cord region was chosen because the noxious heat stimulus in the ‘predictable pain’ data was applied to a region of the palm of the right hand, which corresponds to the C6 dermatome. The extent of these regions and their expected locations were compiled from several anatomical atlases and published papers [24,25,26,27,28]. Each ROI was functionally divided into 7 clusters based on the voxel time-series using k-means clustering, resulting in 70 clusters in total (i.e., 10 × 7). This method provides greater spatial precision by dividing the clusters based on their functional characteristics. All analyses used the same ROIs, and the same cluster definitions established in the ‘predictable pain’ data were used in the ‘resting state’ data, to be able to carry out comparisons between the two data sets.

#### 2.4.1. Structural Equation Modeling (SEM)

Structural equation modeling (SEM) was used as in previous studies [2,7,8,23,29] to examine potential coordinated networks, as cluster-to-cluster correlations may not always sufficiently explain more complex coordination between regions [7,8]. In these studies we have successfully applied SEM to identify and characterize robust resting-state networks in the brainstem and spinal cord [7,8] as well as to characterize connectivity networks during pain processing [9].

**Figure 3:**
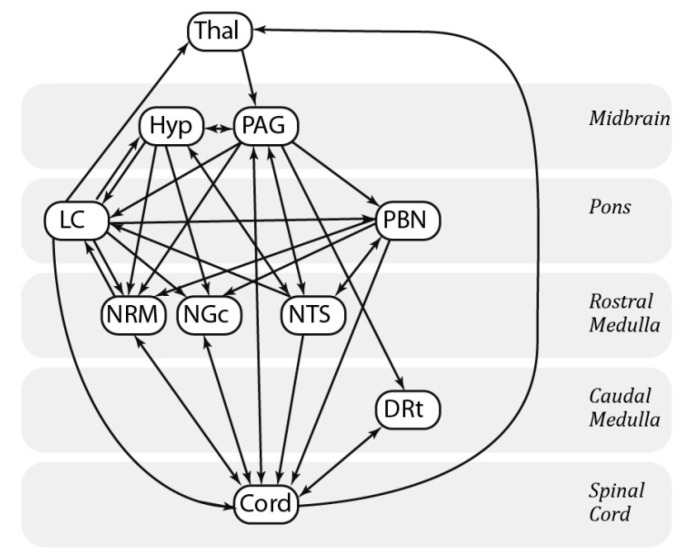
Anatomical model of the regions and connections used for the structural equation modelling (SEM) analysis.

As SEM requires a pre-defined model of anatomical connections to constrain the number of possible results, we have chosen a previously-described model based on known pain-related neuroanatomy [10] and have supplemented it with several possible connections to and from the LC that have been identified in previous animal and physiology studies (Figure 3) [30,31,32].

This model also provides information about the directionality of the modelled connections. For the current study, the 10 brainstem regions described above were used in conjunction with the right dorsal quadrant of the sixth cervical cord segment.

SEM was carried out by means of a general linear model (GLM) to calculate the linear weighting factors (β, the relative contribution of each input to a region), which reflect connectivity between regions. This was done separately for the ‘expectation’ period (between 1 and 2 min into a run, after participants were told whether a stimulus would be applied or not, but before any stimulation occurred) and the ‘stimulation’ period (from 2 min to 2 min 30 s into the run, while participants were either experiencing a noxious stimulus in the ‘pain’ runs, or not experiencing any stimulus in the ‘no pain’ runs). If region A receives input signaling from regions B and C, and the BOLD signal time-series responses in these regions are *S_A_*, *S_B_*, and *S_C_* respectively, then *S_A_ = β_AB_ S_B +_ β_AC_ S_C +_ e_A_* where *e_A_* is the residual signal variation that is not explained by the fit [29]. The model used was divided into several network components, which consisted of multiple ‘source’ regions (e.g., *S_B_*, *S_C_*) providing input to one ‘target region’ (e.g., *S_A_*). The weighting factors (*β*) were calculated separately for each network component, and networks were investigated for every combination of anatomical sub divisions of each region (i.e., the clusters defined above) in order to identify the sub-divisions that resulted in the best fits to the data measured. With this process the β-value for each connection is calculated multiple times, with different combinations of ‘source’ regions and clusters in the same network component.

To determine the goodness-of-fit, the amount of variance in each target region that is explained by the fit was calculated and expressed as an R^2^ value. The significance of the fit was estimated by converting R values to a Z-score by means of Fisher’s Z-transform. The fitting was repeated with one source region at a time omitted from the network in order to identify any terms that did not uniquely account for a significant component of the variance in each target region (computed with an F-test). A cutoff value of F(1,∞) > 3.845 was used, which corresponds to *p* < 0.05. Any terms that did not account for a significant component of the variance were not included in the results.

The significance of each network component was determined based on previously established probability distributions of Z-scores [29], which depend on the model parameters, with significance thresholds set to account for the family-wise error rate of p_fwe_ < 0.05. The significance of β-values was also determined based on their estimated standard errors across participants. Significance was inferred at a family-wise-error corrected p_fwe_ < 0.05 which accounted for the total number of network combinations that were tested across combinations of anatomical sub-divisions.

#### 2.4.2. Analysis of Connectivity Networks in the Predictable Pain Data

Any connections with significant Z-scores in at least one person, for each time period, were then used for a subsequent analysis of covariance (ANCOVA). To do so, the pain sensitivity was calculated for each participant by dividing the average of their peak pain ratings across all ‘stimulation’ runs by the average temperature in degrees Celsius applied to the participant’s hand to elicit that pain (i.e., pain rating/temperature). In this way, participants that experienced moderate pain at lower temperatures elicited higher pain sensitivity than participants that experienced the same pain rating at higher temperatures. An ANCOVA was then applied to the β-values of each participant as the dependent variable, while individual pain sensitivity was used as a continuous independent variable and the study condition (‘pain’ and ‘no pain’) was used as a discrete independent variable. Significance was inferred at a false discovery rate controlled p_FDR_ < 0.05. This analysis identifies the significance of the main effect of study condition (‘pain’ or ‘no pain’), the main effect of pain sensitivity, and the interaction effects between study condition and pain sensitivity.

#### 2.4.3. Comparison of Predictable Pain and Resting State Data

Connections with significant weighting factors were identified for both the ‘expect pain’ and the ‘expect no pain’ groups and were compared with a paired-sample T-test separately for the ‘expectation’ and ‘stimulation’ time periods. Significant differences were inferred at a family-wise-error corrected p_fwe_ < 0.05, which accounted for the total number of comparisons made between the groups. All individual connections that showed significant differences between the ‘expect pain’ and ‘expect no pain’ groups, for both time periods, were then used as a basis for the selection of connections to analyze in the ‘resting state’ data. For example, if β values were significantly different between the ‘expect pain’ and ‘expect no pain’ groups for the connection of PAG cluster 1 to C6RD cluster 4, a comparison was then carried out between the ‘picture’ and ‘video’ conditions of the ‘resting state’ data for the same specific connection. This served as an indicator of potential fluctuations in the resting state due to increased attention [8]. In this way, two groups of connections were identified: connections common to ‘predictable pain’ and ‘resting state’ data (where the connectivity varied significantly both with expectation or experience of pain and resting state fluctuations), and connections unique to the ‘predictable pain’ data (where connectivity varied significantly with the expectation/experience of pain, but not with resting state fluctuations).

## 3. Results

The results of SEM analyses identified significantly connected networks between and within brainstem and spinal cord regions, in both the ‘pain’ and ‘no pain’ conditions of the ‘predictable pain’ data, for both the ‘expectation’ and ‘stimulation’ time periods. The connectivity weighting factors of the connections identified by the SEM analysis were used as the basis for the subsequent ANCOVA analyses. As the ANCOVA analyses are the focus of these results, the intermediary individual SEM results are not presented here.

### 3.1. Analysis of Connectivity Networks in the Predictable Pain Data

**Figure 4:**
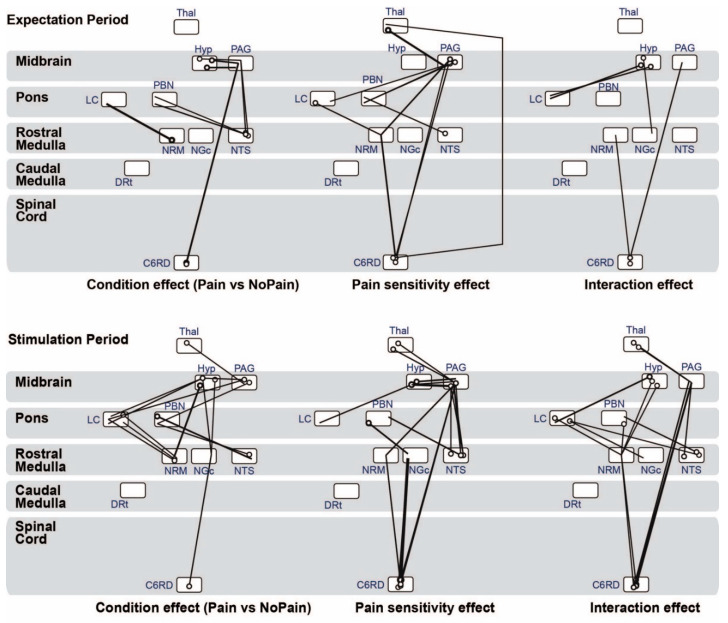
Results of ANCOVA analysis of significant β-values for the ‘expectation’ time period (upper row, after participants knew whether or not to expect pain, but before a stimulus was applied) and ‘stimulation’ time period (lower row, while participants were either experiencing pain or no stimulus). Pictured from left to right are the connections with a significant main effect of condition (‘pain’, where a stimulus would be applied, and ‘no pain’, where no stimulus would be applied), a significant main effect of individual pain sensitivity, and significant condition × sensitivity interaction effects. Significant connections are shown at a false-discovery-rate corrected p_FDR_ < 0.05. Lines represent significant connections, circles represent the connection’s source, and the line end represents the target region. Rectangular bubbles represent anatomical regions, and the space inside the bubble is divided into the region’s different clusters. Only the C6RD segment of the spinal cord is used as being representative of brainstem-cord connectivity, because it corresponds to the heat stimulus applied to the hand.

The analysis of covariance (ANCOVA) revealed that there are significant variations in brainstem and spinal cord connectivity in relation to the condition (‘pain’ or ‘no pain’), and in relation to individual pain sensitivity. These results for the main effects of condition and individual pain sensitivity, as well as condition x pain sensitivity interaction effects are shown in Figure 4.

During the ‘expectation’ time period, before a stimulus was applied, connectivity strengths varied significantly between conditions for the connections from the hypothalamus, NTS, and spinal cord to the PAG, from the NTS to the PBN, and from the NRM to the LC. The connectivity strength also varied significantly in relation to individual pain sensitivity for the connections from the PAG to the LC, PBN, NRM, and spinal cord, from the spinal cord to the NRM and thalamus, from the LC to the NRM, from the NTS to the PBN, and from the thalamus to the PAG.

During the ‘stimulation’ period (during which participants either felt the painful stimulus, or no stimulus in the ‘no pain’ condition) connectivity strengths varied significantly between conditions for the connections from the NRM to the LC, from the hypothalamus to the PAG, from the NTS to the PAG and PBN, and from the spinal cord to the PAG. Connectivity strengths also varied significantly with individual pain sensitivity for the connections from the thalamus to the PAG, from the PAG to the LC, PBN, and NRM, from the NTS to the PBN, and from the spinal cord to the NRM, PAG, and thalamus. Additionally, connectivity from the hypothalamus to LC and NGc, and from the spinal cord to PAG and NRM connections showed significant condition x pain sensitivity interaction effects for both the ‘expectation’ and ‘stimulation’ time periods.

### 3.2. Comparison of Predictable Pain and Resting State Data

**Figure 5:**
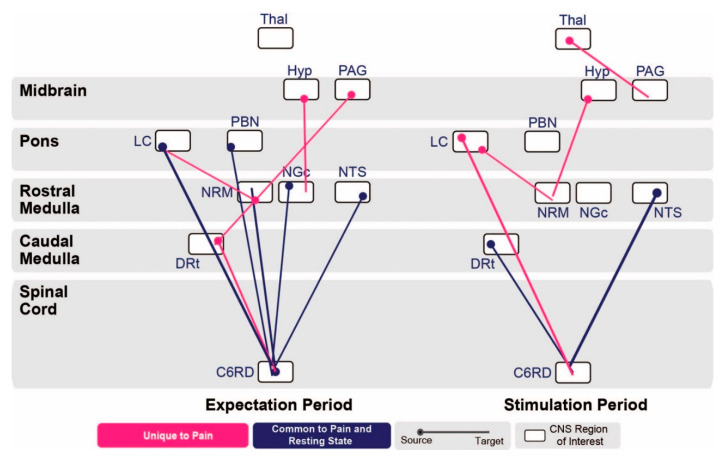
Connections with significant differences in β-values between the ‘pain’ and ‘no pain’ conditions of the ‘predictable pain’ data, contrasted with connections with significant differences in β-values between the ‘picture’ and ‘video’ conditions of the ‘resting state’ data, by the use of paired-sample T-tests. Pictured are the comparison of connections in the ‘expectation’ period (left) and ‘stimulation’ period (right). Connections marked in dark blue represent connections with differences in β-values common to both ‘expecting pain’ and ‘resting state’ (β-values fluctuate significantly with the expectation/experience of pain but also due to resting-state fluctuations). Lines represent significant connections, the circles represent the connection’s source, and the line end represents the connection’s target region. Rectangular bubbles represent anatomical regions, and the space inside the bubble is divided into the region’s different clusters. Connections marked in light pink represent connections with differences in β-values unique to ‘expecting pain’ (β-values fluctuate significantly with the expectation/experience of pain, but not resting-state fluctuations). Significant connections are shown at a family-wise error rate corrected p_fwe_ < 0.05. Only the C6RD segment of the spinal cord is used as being representative of brainstem-cord connectivity because it corresponds to the dermatome where the heat stimulus was applied to the hand.

To further examine these networks, we compared connections with significantly different β-values between the ‘pain’ and ‘no pain’ conditions, to resting state data during the ‘picture’ and ‘video’ conditions (Figure 5).

During the ‘expectation’ period, several connections showed variations in connectivity that were common between the ‘predictable pain’ and ‘resting state’ data. Namely, they had significantly different β-values both when comparing the ‘pain’ and ‘no pain’ conditions, and when comparing the ‘video’ and ‘picture’ conditions. These connections were from the LC, PBN, NGc, and NTS to the spinal cord, and from the spinal cord to the NRM. In contrast, several connections were unique to ‘predictable pain’ (where connectivity varied significantly with the expectation/experience of pain, but not with resting state fluctuations). The connections with significant β-value variation unique to expecting pain were from the hypothalamus to the NGc, from the PAG to the DRt, from the NRM to the LC, and from the DRt to the spinal cord.

During the ‘stimulation’ period, the connections identified as common to ‘predictable pain’ and ‘resting state’ data were from the DRt and NTS to the spinal cord, while connections identified as unique to ‘predictable pain’ data were from the thalamus to the PAG, from the hypothalamus to the NRM, and from the LC to the NRM and spinal cord.

## 4. Discussion

Our prior studies have provided evidence of coordinated resting-state networks in the brainstem and spinal cord, and coordinated descending regulation during the anticipation and experience of pain. The results of the present study now reveal aspects of the relationships between resting-state networks and pain regulation. We first compared brainstem and spinal cord network connectivity during ‘pain’ and ‘no pain’ in the ‘predictable pain’ data, including in relationship to individual pain sensitivity. During both the ‘expectation’ and ‘stimulation’ periods, ANCOVA results showed specific variations in brainstem and spinal cord connectivity in relation to both the study condition and individual pain sensitivity (Figure 4). We identified several connections with connectivity that varied significantly with the study condition (‘pain’ or ‘no pain’); namely, from the hypothalamus and NTS to the PAG, from the NTS to the PBN, and from the NRM to the LC. Similarly, connectivity from the thalamus to PAG, PAG to LC, PBN, and NRM, spinal cord to NRM and thalamus, and NTS to PBN, varied with individual pain sensitivity regardless of whether participants were expecting or experiencing pain. Many of these connectivity differences which relate to pain sensitivity were predictably stronger during the ‘stimulation’ period. There were also interaction effects observed in connections from the hypothalamus to the LC and NGc, and from the spinal cord to the NRM and PAG, and all were consistent between the ‘expectation’ and ‘stimulation’ time periods.

These networks include regions involved in the descending modulation of pain, as well as other areas that are part of a previously-described larger network [10,30,32], and include areas that are involved in some motivational-affective components of pain [31]. These effects are continuous across the two time periods analyzed in the experiment and are not specific to the experience of a painful stimulus. Similar results have been described in a previous study that outlined continuous pain modulation across time, independent of whether a participant was feeling pain [9]. Connectivity variations in many of these regions (including the hypothalamus, NTS, PBN, and LC) were also described in a previous resting-state study, where differences in a participant’s attention likely drove the effects [8]. In this case, however, it is unlikely that differences in attentiveness were the primary driver of these continuous effects, as we see connectivity vary specifically with an individual’s pain sensitivity. Pain sensitivity has also been shown to explain pain related outcomes [33] in chronic pain situations when controlling for other factors, further supporting the idea that connectivity variations with individual pain sensitivity could point to differences in overall pain regulation. It is possible that these effects are due in part to differences in salience of the pain stimulus, as those individuals with high pain sensitivity may perceive a painful stimulus as more salient than those with a lower pain sensitivity. These results indicate that these effects are at least in part linked to continuous pain modulation at the level of the brainstem and spinal cord.

In addition to the connections with similar connectivity variations in both the ‘expectation’ and ‘stimulation’ epochs, we also identified connections with variations that only occurred in specific situations. The PAG to spinal cord connection showed differences in connectivity in relation to pain sensitivity, but not to the study condition, and this effect was limited to the ‘expectation’ time period. In contrast, during the ‘stimulation’ period, connectivity from the spinal cord to the PAG showed a main effect of the study condition and pain sensitivity but had no significant interaction effects. This shows that connectivity varied independently with a participant’s pain sensitivity as well as with which study condition they were experiencing. These results may indicate that the PAG to spinal cord connectivity is involved in regulatory pain modulation that occurs before a stimulus is felt, while spinal cord to PAG connectivity is part of a more specific feedback mechanism that occurs as a result of noxious input processed at the level of the spinal cord, and is regulated by brainstem areas.

To further explore these networks, we compared connections with significant weighting factors for the ‘pain’ and ‘no pain’ conditions in both time periods, to ‘resting state’ data during the ‘picture’ and ‘video’ conditions (Figure 5). This was done in order to assess which differences in network connectivity occur when participants are expecting and experiencing pain, and to compare these effects to differences in connectivity that can occur due to resting state or attention-driven fluctuations. During the ‘expectation’ period, differences between the ‘pain’ and ‘no pain’ conditions involve network connectivity fluctuations that were also previously described in resting state networks [7,8]. Differences in connectivity from the LC, PBN, NGc, and NTS to the spinal cord, and from the spinal cord to the NRM were common to both the expectation of pain and resting state effects. During the ‘stimulation’ period, connectivity differences from the DRt and NTS to the spinal cord were also common between experiencing pain and resting state effects. These commonalities involve altered connectivity to and from the spinal cord, which may be evidence of a spinal cord resting state comparable to the default mode network in the brain. This idea is supported by the fact that these networks are still observed in part during the ‘stimulation’ period, similar to how activity in the default mode network is still observed but decreased during a stimulus or task [34].

We also observed connectivity differences between the ‘pain’ and ‘no pain’ conditions in the ‘expectation’ period, which were not present when comparing a resting state (‘picture’) to an altered cognitive state (‘video’), shown in Figure 5. This included connections from the hypothalamus to the NGc, from the PAG to the DRt, from the NRM to the LC, and from the DRt to the spinal cord. These differences in network connectivity therefore seem to be unique to expecting pain, and include regions involved in descending pain modulation [10,35,36] such as the PAG, NRM, NGc, and spinal cord. During the ‘stimulation’ period, connectivity in these regions as well as the thalamus also showed unique variations not found in the resting state effects. The PAG-RVM pathway has been found to be involved in producing analgesia in situations of threatened pain, but inhibited when there was no threat of pain [35,36], further supporting the idea that these effects are linked specifically to the expectation of pain.

The variations observed to be specific to the expectation of pain also involve distinct brainstem-to-brainstem connectivity in areas such as the hypothalamus, LC, NRM, and DRt, which may be related to homeostatic autonomic regulation as part of a larger network of integrated functions [7,37,38,39]. Cognitive processes such as attention can also influence autonomic activity [39], and participants are likely more attentive when they are expecting to feel pain as opposed to waiting for the study to end. A system including areas such as the hypothalamus, NTS, RVM (all of which showed distinct connectivity differences in the ‘expectation’ period) was previously described as part of a homeostatic afferent network [37].

This suggests that connectivity during the expectation of pain involves network elements related to pain modulation and autonomic homeostatic regulation. It is unlikely that these changes are driven primarily by a difference in alertness, because some of these modulatory changes in connectivity are present during the ‘stimulation’ period as well and vary significantly with individual pain sensitivity. This shows that continuous pain modulation carries on as participants are experiencing pain, supporting the conclusion that these results represent pain regulation before a stimulus is felt. While some of these effects could be driven in part by differences in salience, current evidence suggests there is more at play. The network components identified here include pathways known to be involved in descending pain modulation [10,35,36], homeostatic regulation [37,38,39], and pathways that have been shown to produce analgesia in situations of threatened pain [35,36]. Therefore, the differences unique to the ‘expectation’ period involve pain modulation, and this pain modulation occurs even before a stimulus is applied. Previous spinal cord fMRI studies that have specifically focused on these effects by analyzing BOLD signal responses to a stimulus showed that a state of threat or safety can alter descending modulation of pain [40,41,42]. However, it is also important to consider how activity across brainstem and spinal cord regions is linked to the expectation of pain itself. The consistency of current and past results supports the conclusion that coordinated brainstem and spinal cord networks are altered in specific ways during the expectation of pain, and that continuous pain modulation is an important effect to consider even in baseline periods before a painful stimulus is applied.

## 5. Conclusions

The current study examined data from participants expecting and experiencing pain and compared them to data of participants experiencing a resting state or engaging video. Results showed extensive connectivity within/across BS and SC regions during the expectation of pain, and components of these networks varied significantly with individual pain sensitivity. Comparing these results to resting-state network fluctuations revealed that while some of these network fluctuations are common to resting-state effects, elements of these networks are unique to the expectation of pain. The regions involved in these unique elements provide evidence of brainstem pain regulation during the expectation of pain.
